# MicroRNA-146a: A Key Regulator of Astrocyte-Mediated Inflammatory Response

**DOI:** 10.1371/journal.pone.0044789

**Published:** 2012-09-13

**Authors:** Anand Iyer, Emanuele Zurolo, Avanita Prabowo, Kees Fluiter, Wim G. M. Spliet, Peter C. van Rijen, Jan A. Gorter, Eleonora Aronica

**Affiliations:** 1 Department of (Neuro) Pathology, Academic Medical Center, University of Amsterdam, Amsterdam, The Netherlands; 2 Department of Genome Analysis, Academic Medical Center, University of Amsterdam, Amsterdam, The Netherlands; 3 Department of Pathology, University Medical Center Utrecht, Utrecht, The Netherlands; 4 Department of Neurosurgery/Rudolf Magnus Institute for Neuroscience, University Medical Center Utrecht, Utrecht, The Netherlands; 5 Swammerdam Institute for Life Sciences, Center for Neuroscience, University of Amsterdam, Amsterdam, The Netherlands; 6 SEIN-Epilepsy Institute in the Netherlands Foundation, Heemstede, The Netherlands; Institute Biomedical Research August Pi Sunyer (IDIBAPS) - Hospital Clinic of Barcelona, Spain

## Abstract

Increasing evidence supports the involvement of microRNAs (miRNA) in the regulation of inflammation in human neurological disorders. In the present study we investigated the role of miR-146a, a key regulator of the innate immune response, in the modulation of astrocyte-mediated inflammation. Using Taqman PCR and in situ hybridization, we studied the expression of miR-146a in epilepsy-associated glioneuronal lesions which are characterized by prominent activation of the innate immune response. In addition, cultured human astrocytes were used to study the regulation of miR-146a expression in response to proinflammatory cytokines. qPCR and western blot were used to evaluate the effects of overexpression or knockdown of miR-146a on IL-1β signaling. Downstream signaling in the IL-1β pathway, as well as the expression of IL-6 and COX-2 were evaluated by western blot and ELISA. Release several cytokines was evaluated using a human magnetic multiplex cytokine assay on a Luminex® 100™/200™ platform. Increased expression of miR-146a was observed in glioneuronal lesions by Taqman PCR. MiR-146a expression in human glial cell cultures was strongly induced by IL-1β and blocked by IL-1β receptor antagonist. Modulation of miR-146a expression by transfection of astrocytes with anti-miR146a or mimic, regulated the mRNA expression levels of downstream targets of miR-146a (IRAK-1, IRAK-2 and TRAF-6) and the expression of IRAK-1 protein. In addition, the expression of IL-6 and COX-2 upon IL-1β stimulation was suppressed by increased levels of miR-146a and increased by the reduction of miR-146a. Modulation of miR-146a expression affected also the release of several cytokines such as IL-6 and TNF-α. Our observations indicate that in response to inflammatory cues, miR-146a was induced as a negative-feedback regulator of the astrocyte-mediated inflammatory response. This supports an important role of miR-146a in human neurological disorders associated with chronic inflammation and suggests that this miR may represent a novel target for therapeutic strategies.

## Introduction

During the last decade, both experimental and clinical studies have demonstrated the relevance of inflammation in the pathophysiology of epilepsy (for reviews see [1]–[3]. Neuropathological examination of surgical epilepsy specimens provides evidence of a complex and sustained inflammatory phenomenon with production of pro-inflammatory molecules in patients with temporal lobe epilepsy (TLE) with hippocampal sclerosis (HS). Activation of cells of the microglia/macrophage lineage and astrocytes, and concomitant induction of various inflammatory pathways, also have been observed in focal malformations of cortical development (MCD; such as focal cortical dysplasia FCD), which represent major causes of pediatric epilepsy [4]. Both the innate and the adaptive immune responses are activated in these lesions ([5]; reviewed in [3]). In particular, both in vitro and in vivo data, point to the role of astrocytes as a major source and/or targets of pro-epileptogenic inflammatory signaling, such as the interleukin(IL)-1, High Mobility Group Box 1 (HMGB1) and Toll-like receptor (TLR) signaling pathways [1], [3], [6]–[9].

An increasing body of literature supports the critical role of miRNAs in post-transcriptional gene regulation in several biological processes of the central nervous system (CNS), as well as in the pathogenesis of different disorders (including developmental, neurodegenerative, vascular and neuroinflammatory disorders), and in oncogenesis [10]–[12]. Various miRNAs are also considered to represent a new class of mediators of inflammation [13]–[16]. In particular, miRNA-146a has been associated with the regulation of Toll-like and interleukin-1 receptors (TIRs) signaling [15], [17]–[19]. Interestingly, this miRNA has been shown to be upregulated in experimental models of epilepsy, as well as in human TLE [20]–[22]. miRNA-146a is expressed in human brain in astrocytes which may be key targets in the regulation of this miRNA in response to inflammatory molecules [19], [20].

In the present study we evaluated the role of miR-146a in human astrocytes. We first assessed the expression levels of miR-146a in epilepsy-associated glioneuronal lesions (ganglioglioma, GG; focal cortical dysplasia, FCD type IIb) characterized by prominent activation of the innate immune response in astroglial cells [3], [23]–[25] and analyzed the cellular distribution in FCD type IIb. Furthermore, we investigated the regulation of miR-146a in response to inflammatory molecules in cultured human glial cells (U373 glioblastoma cell line and human astrocytes cell cultures) and further evaluated its role in regulating TLR/IL-1R-interleukin-1 receptor-associated kinase (IRAK)-NF-κB signaling, particularly in astroglia.

## Materials and Methods

### Human Material

The cases included in this study were obtained from the archives of the departments of neuropathology of the Academic Medical Center (University of Amsterdam) and the University Medical Center in Utrecht. A total of 6 brain tissue specimens, removed from patients undergoing surgery for intractable epilepsy, were examined. Informed written consent was obtained for the use of brain tissue and for access to medical records for research purposes. Tissue was obtained in accordance with the Declaration of Helsinki and the AMC Research Code provided by the Medical Ethics Committee of the AMC. All cases were reviewed independently by two neuropathologists and the diagnosis was confirmed according to the international consensus classification system recently proposed for grading FCD [23]. For the GG we used the revised WHO classification of tumors of the central nervous system [26]. **Table S1** summarizes the clinical findings of epilepsy patients and controls. None of the FCD patients fulfilled the diagnostic criteria for tuberous sclerosis complex (TSC). All patients underwent presurgical evaluation with investigations consisting of non-invasive tests, including history, medical, neurological and neuropsychological assessment, structural neuroimaging and extensive interictal and ictal electroencephalographic (EEG) studies with video monitoring. Patients who underwent implantation of strip and/or grid electrodes for chronic subdural invasive monitoring before resection were excluded from this study. Patients had complex partial seizures (CPS) and all patients had daily seizures, which were resistant to maximal doses of anti-epileptic drugs. Seizure duration represents the interval in years from age at seizure onset and age at surgery. Normal-appearing control cortex was obtained at autopsy from 6 age-matched patients without history of seizures or other neurological diseases. Autopsied brain tissues from patients with neuro-inflammatory pathologies (viral encephalitis; herpes simplex encephalitis and [27] and rabies encephalitis [28]) were also examined as positive controls). All autopsies were performed within 12 hours after death. Furthermore, we also used histologically normal temporal neocortex from patients undergoing extensive surgical resection of the mesial structures for the treatment of medically intractable complex partial epilepsy to control for a possible effect of recurrent seizures, antiepileptic drugs or post mortem interval on the expression of miR146a.

### Tissue Preparation

Brain tissue from control patients and surgical tissue block from patients with epilepsy was snap frozen in liquid nitrogen and stored at −80°C until further use (RNA isolation for RT-PCR). Additional tissue was fixed in 10% buffered formalin and embedded in paraffin. Paraffin-embedded tissue was sectioned at 6 µm, mounted on pre-coated glass slides (Star Frost, Waldemar Knittel GmbH, Brunschweig, Germany) and two sections were used for in situ hybridizations and immunocytochemistry. Two additional sections were used for the double staining, combining in situ hybridization with immunocytochemistry (in the same sections) with different antibodies, as described below. One representative paraffin block per case (containing the complete lesion or the largest part of the lesion resected at surgery) were sectioned, stained and assessed. Sections of all specimens were processed for haematoxylin eosin (HE), luxol fast blue (LFB) and Nissl stains, as well as for immunocytochemical stainings for a number of neuronal and glial markers to confirm the diagnosis of FCD IIb.

### In situ Hybridization

In situ hybridization for miR-146a was performed using a 5′ fluorescein labeled 19 mer antisense oligonucleotide containing Locked Nucleic Acid (LNA) and 2′OME RNA moieties (FAM - AacCcaTggAauTcaGuuCucA, capitals indicate LNA, lower case indicates 2′OME RNA). The oligo’s were synthesized by Ribotask ApS, Odense, Denmark. The hybridizations were done on 6 µm sections of paraffin embedded materials described previously [29]. The hybridization signal was detected using a rabbit polyclonal anti-fluorescein/Oregon green antibody (A21253, Molecular probes, Invitrogen) and a horse radish peroxidase labeled goat anti-rabbit polyclonal antibody (P0448 Dako, Glostrup Denmark) as secondary antibody. Signal was detected with chromogens 3-amino-9-ethyl carbazole (St. Louis, MO, USA) or Vector NovaRed (Vector Laboratories, Burlingame, CA, USA) and the nuclei were stained with haematoxylin. Slides were sealed with glycerol-gelatin (St. Louis, MO, USA). Sections without probe or with scramble probes were blank. For the double staining, combining immunocytochemistry with in situ hybridization, sections were first processed for immunocytochemistry as previously described [24] with GFAP (glial fibrillary acidic protein; polyclonal rabbit, DAKO, Glostrup, Denmark; 1∶4000) or HLA-DR (anti-human leukocyte antigen (HLA)-DP, DQ, DR (mouse clone CR3/43; DAKO, Glostrup, Denmark; 1∶400) using Fast Blue B salt (St. Louis, MO, USA) or Vector Blue substrate (Vector Laboratories, Burlingame, CA, USA) as chromogen. After washing, sections were processed for in situ hybridization as described above. Images were captured with an Olympus microscope (BX41, Tokyo, Japan) equipped with a digital camera (DFC500, Leica Microsystems-Switzerland Ltd., Heerbrugg, Switzerland).

### RNA Isolation

For RNA isolation, 800 µl Trizol LS Reagent (Invitrogen, Carlsbad, CA, USA) was added to 0.1–0.5×10^6^ cells. After addition of 200 µg glycogen and 200 µl chloroform, the aqueous phase was isolated using Phase Lock tubes (5 Prime GmBH, Hamburg, Germany). RNA was precipitated with isopropyl alcohol, washed with 75% ethanol and dissolved in water. The concentration and purity of RNA were determined at 260/280 nm using a nanodrop spectrophotometer (Thermo Fisher Scientific, Wilmington, DE, USA).

### Real-time Quantitative PCR Analysis (qPCR)

Five micrograms of total RNA were reverse-transcribed into cDNA using oligo dT primers. Five nmol oligo dT primers were annealed to 5 µg total RNA in a total volume of 25 µl, by incubation at 72°C for 10 min, and cooled to 4°C. Reverse transcription was performed by the addition of 25 µl RT-mix, containing: First Strand Buffer (Invitrogen-Life Technologies), 2 mM dNTPs (Pharmacia, Germany), 30 U RNAse inhibitor (Roche Applied Science, Indianapolis, IN, USA) and 400 U M-MLV reverse transcriptase (Invitrogen - Life Technologies, The Netherlands). The total reaction mix (50 µl) was incubated at 37°C for 60 min, heated to 95°C for 10 min and stored at −20°C until use.

PCR primers (Eurogentec, Belgium) were designed using the Universal Probe Library of Roche (https://www.roche-applied-science.com) on the basis of the reported mRNA sequences. For the human cell cultures we used the following primers: IRAK1 (forward: gcccgaggagtacatcaaga; reverse: ctctgaccagccaaggtctc), IRAK2 (forward: cctcctctgaggcctgtgt; reverse: tgatctcaatttgccacgaa), TNF receptor associated factor 6 (TRAF6; forward: tggcattacgagaagcagtg; reverse: tggacatttgtgacctgcat:), interleukin 6 (IL-6; forward: ctcagccctgagaaaggaga; reverse: tttcagccatctttggaagg), cyclooxygenase-2 (COX-2; forward: gaatggggtgatgagcagtt; reverse: gccactcaagtgttgcacat), elongation factor 1-alpha (EF1a; foward: atccacctttgggtcgcttt; reverse: ccgcaactgtctgtctcatatcac) and hypoxanthine phosphoribosyl transferase (HPRT; forward: tggcgtcgtcgtgattagtgatg; reverse: tgtaatccagcaggtcagca). For each PCR, a mastermix was prepared on ice, containing per sample: 1 µl cDNA, 2.5 µl of FastStart Reaction Mix SYBR Green I (Roche Applied Science, Indianapolis, IN, USA), 0.4 µM of both reverse and forward primers. The final volume was adjusted to 5 µl with H_2_O (PCR grade). The LightCycler® 480 Real-Time PCR System (Roche-applied-science) was used with a 384-multiwell plate format. The cycling conditions were carried out as follows: initial denaturation at 95°C for 5 min, followed by 45 cycles of denaturation at 95°C for 15 s, annealing at 55–60°C for 5 s and extension at 72°C for 10 s. The fluorescent product was measured by a single acquisition mode at 72°C after each cycle. For distinguishing specific from non-specific products and primer dimers, a melting curve was obtained after amplification by holding the temperature at 65°C for 15 s followed by a gradual increase in temperature to 95°C at a rate of 2.5°C s^−1^, with the signal acquisition mode set to continuous [30]. Quantification of data was performed using the computer program LinRegPCR in which linear regression on the Log(fluorescence) per cycle number data is applied to determine the amplification efficiency per sample [31]. The starting concentration of each specific product was divided by the starting concentration of reference genes (EF1a and HPRT) and this ratio was compared between patient/control groups.

miRNA (miR-146a and the U6B small nuclear RNA gene, rnu6b) expression was analyzed using Taqman microRNA assays (Applied Biosystems, Foster City, CA). cDNA was generated using Taqman MicroRNA reverse transcription kit (Applied Biosystems, Foster City, CA) according to manufacturer’s instructions and the PCRs were run on a Roche Lightcycler 480 (Roche Applied Science, Basel, Switzerland), according to the instructions of the manufacturer. Data analysis was performed with the software provided by the manufacturer.

### Cell Cultures

For astrocytes-enriched human cell cultures, fetal brain tissue (15–23 weeks of gestation) was obtained from spontaneous or medically induced abortions with appropriate maternal written consent for brain autopsy. Tissue was obtained in accordance with the Declaration of Helsinki and the AMC Research Code provided by the Medical Ethics Committee of the AMC. Resected tissue samples were collected in Dulbecco’s modified Eagle’s medium (DMEM)/HAM F10 (1∶1) medium (Gibco, Life Technologies), supplemented with 50 units/ml penicillin and 50 µg/ml streptomycin and 10% fetal calf serum (FCS). Cell isolation was performed as previously described [32], [33]. Briefly, after removal of meninges and blood vessels, tissue was minced and dissociated by incubation at 37°C for 20 min in a Hank’s balanced salt solution containing 2.5 mg/ml trypsin (Sigma, St. Louis, MO, USA) and 0.1 mg/ml bovine pancreatic Dnase I (Boehringer Mannheim, Germany). Tissue was triturated and washed with DMEM/HAM F10 medium, supplemented with 50 units/ml penicillin and 50 µg/ml streptomycin and 10% FCS. Cell suspension (containing ∼ 0.5 g wet weight tissue/10 ml culture medium) was passed through a 70 µm cell sieve (Becton Dickinson, USA) and plated into poly-L-lysine (PLL; 15 µg/ml, Sigma) pre-coated 25 cm^2^ flasks (Falcon, Lincoln Park, NJ) and maintained in a 5% CO_2_ incubator at 37°C. After 48 h the culture medium was replaced by fresh medium and cultures were subsequently refreshed twice a week. Cultures reached confluence after 2–3 weeks. Secondary astrocyte cultures were established by trypsinizing confluent cultures and sub-plating onto PLL-precoated 6 and 24-well plates (Costar; 0.5×10^6^ cell/well in a 6-well plate for western blot analysis or 0.1×10^6^ cell/well in a 24-well plate for RNA isolation and PCR) and simultaneously into PLL- precoated 12 mm coverslips (Sigma) in 24-well plates (Costar; 2×10^4^ cell/well; for immunocytochemistry). More than 98% of the cells in primary culture, as well as in the successive 12 passages were strongly immunoreactive for the astrocytic marker GFAP and S100β. In the present study astrocytes were used for immunocytochemical analyses at passage 3–4.

The astrocytoma cell line U373 was obtained from the American Type Culture Collection (Rockville, MD, USA); cells were cultured in (DMEM)/HAM F10 (1∶1) supplemented with 50 units/ml penicillin, 50 µg/ml streptomycin and 10% FCS.

### Treatment of Cell Cultures

Human recombinant (r)IL-1ß (Peprotech, NJ, USA; 10 ng/ml) was applied and maintained for different time periods (from 10 min to 48 h) before harvesting the cells for RNA isolation or western blot analysis. Medium was collected to perform enzyme-linked immunosorbent assay (ELISA). In some experiments lipo-polysaccharide (LPS; 100 ng/ml; Sigma, St. Louis, USA), rIL-6 (10 ng/ml; Strathmann Biotec A.G., Hamburg, Germany), tumor necrosis factor α (TNFα; 1 ng/ml; Peprotech, NJ, USA) and High mobility group box 1 (HMGB1; 40 nM; HMGBiotech S.r.l., Milan, Italy) alone or together with IL-1 ß were applied and maintained in the medium for 24 before harvesting the cells for RNA isolation. In some experiments cells stimulated with LPS were treated with LPS-RS (a TLR4 antagonist from the photosynthetic bacterium *Rhodobacter sphaeroides*; Invivogen, Toulouse, France; 10 µg/ml), applied 1 h before LPS. Human IL-1 receptor antagonist (IL-1Ra; 1 µg/ml; Peprotech, NJ, USA) was used to neutralize IL-1β activity (applied 1 h before IL-1β). As previously shown [33], the viability of human astrocytes in culture was not influenced by the treatments.

### Transfection of Cells

Cells in 12-well plates were transfected either with anti-miR146a LNA (FAM – AacCcaTggAauTcaGuuCucA; Ribotask ApS, Odense, Denmark) or scrambled LNA (FAM™ dye-labeled Anti-miR™ Negative Control #1; Applied Biosystems, Carlsbad, CA, USA) in blocking experiments or with miR146a precursor (pre-mir146a; mir146 mimic, Applied Biosystems, Carlsbad, CA, USA) or scrambled pre-mir (FAM™ dye-labeled Pre-miR™ Negative Control #1; Applied Biosystems, Carlsbad, CA, USA) in overexpression experiments using Lipofectamine (Invitrogen, USA) according to manufacturer’s instructions. Briefly, 5 µl lipofectamine was added to 200 µl of serum free medium (DMEM + F10) per condition for 5 minutes followed by incubation with anti-miR146a LNA or pre-miR146a at a final concentration of 50nM. The volume was adjusted to 1 ml with medium containing 10% FCS and added to the cells. Transfection efficiency was found to be around 80% by flow cytometry in both U373 and primary astrocytes. After 24 hours the IL-1β was added to the wells for 24 hours after which the cells were harvested for RNA or protein isolation.

### Preparation of Cellular Extracts and Western Blot Analysis

Cells were harvested at 24 h after treatments and/or transfection. Medium was collected (for the detection of cytokine and HMGB1 release) and glial cells were washed twice with cold PBS. The samples were homogenized in lysis buffer containing 10 mM Tris (pH 8.0), 150 mM NaCl, 10% glycerol, 1% NP-40, Na orthovanadate (10.4 mg/ml), 5 mM EDTA (pH 8.0), 5 mM NaF and protease inhibitor cocktail (Boehringer Mannheim, Germany) by incubating on ice for 15 minutes. The homogenates were centrifuged at 14000 rpm for 10 mins and the supernatant was used for further analysis. Protein content was determined using the bicinchoninic acid method [34]. Western blot analysis was performed, as previously described [35]. For electrophoresis, equal amounts of proteins (15–20 µg/lane) were separated on a 10% sodium dodecylsulfate-polyacrylamide gel electrophoretic (SDS-PAGE) gel. Separated proteins were transferred to nitrocellulose paper for 90 min at 100 V, using a wet electroblotting system (BioRad, Hercules, CA, USA). Blots were blocked for 1 hour in 5% non fat dry milk in Tris-buffered saline-Tween (TBST) (20 mM Tris, 150 mM NaCl, 0.1% Tween 20, pH 7.5; for IRAK1 and IRAK2) or in 5% bovine serum albumin (BSA) in TBST (for TRAF6). The blots were incubated overnight with the primary antibody (in 5% milk solution 1∶1000 IRAK1 rabbit polyclonal antibody; 1∶500 IRAK2 rabbit polyclonal antibody; 1∶500 in 5% BSA solution TRAF6 mouse monoclonal, (all from Santa Cruz, CA, USA); COX-2 (1∶2500, goat anti-human; Cayman Chemical Company, Ann Arbor, MI, USA); HMGB1 (rabbit polyclonal antibody 1∶1000; Pharmingen, San Diego, CA, USA).

After several washes in TBST, the membranes were incubated in TBST/5% non fat dry milk, containing the goat anti-rabbit or rabbit anti-mouse coupled to horse radish peroxidase (1∶2500; Dako, Denmark) for 1 h. After washes in TBST, immunoreactivity was visualized using ECL PLUS western blotting detection reagent (GE Healthcare Europe, Diegen, Belgium). Expression of β-actin (monoclonal mouse, Sigma, St. Louis, MO, 1∶50000) and β-tubulin (1∶30000, monoclonal mouse, Sigma, St. Louis, MO, USA) was used as loading control. For the quantification of the blots the band intensities were measured densitometrically Scion Image for Windows (beta 4.02) image-analysis software. A ratio of the integrated band density (IntDen) of the protein of interest to the IntDen of the reference protein was used to normalize band intensities.

### Detection of Cytokine Release

Evaluation of levels of 16 cytokines/chemokines/inflammatory molecules [IL-1β, IL-5, IL-6, IL-8, IL-10, IL-13, IL-17, IL-18, interferon (IFN)-α2, IFN-γ, IP-10 (CXCL10), macrophage inflammatory protein-1β (MIP-1β), G-CSF (granulocyte-colony stimulating factor), Rantes, Tumor necrosis factor (TNF)-α, and VEGF] in the medium of treated cultures was carried out with the human magnetic 10-plex panel (Invitrogen, Camarillo, CA, USA) to which extra cytokine beads were added according to the manufacturer’s protocol. The plate was read on a Luminex® 100™/200™ platform. Data were analyzed and statistics calculated with GraphPad Prism 4 software (San Diego, CA, USA).

Levels of MCP-1 were measured using a kit from Ebioscience (Vienna, Austria) according to manufacturer’s instructions. Briefly, flat bottomed ELISA microtiter plates (Costar, Cambridge, MA, USA) were coated overnight with the capture antibody in coating buffer. The plates were washed, preblocked with assay diluent (provided with the kit and incubated with the culture supernatants diluted 1∶50 assay diluent or standard antigen dilutions in triplicate. The plates were washed and incubated with biotinylated detection antibody followed by horse radish peroxidase labelled streptavidin. Tetramethyl benzidine (TMB) (Sigma Chemical Co, USA) was used as the colour substrate, and the reaction was stopped with an equal volume of 1 M H_2_SO_4_. The absorbance was read in a Titertek Multiskan microplate reader (Germany) at 450 nm with a reference wavelength of 570 nm.

### Statistical Analysis

Statistical analyses were performed with Graphpad Prism® software (Graphpad software Inc., La Jolla, CA, USA) using two-tailed Student’s t-test or, for multiple groups, a non-parametric Kruskal–Wallis test followed by the Dunn’s post hoc test to assess differences between groups. P<0.05 was considered significant.

## Results

### miR-146a Expression in GG and FCD Type IIb

**Figure 1:**
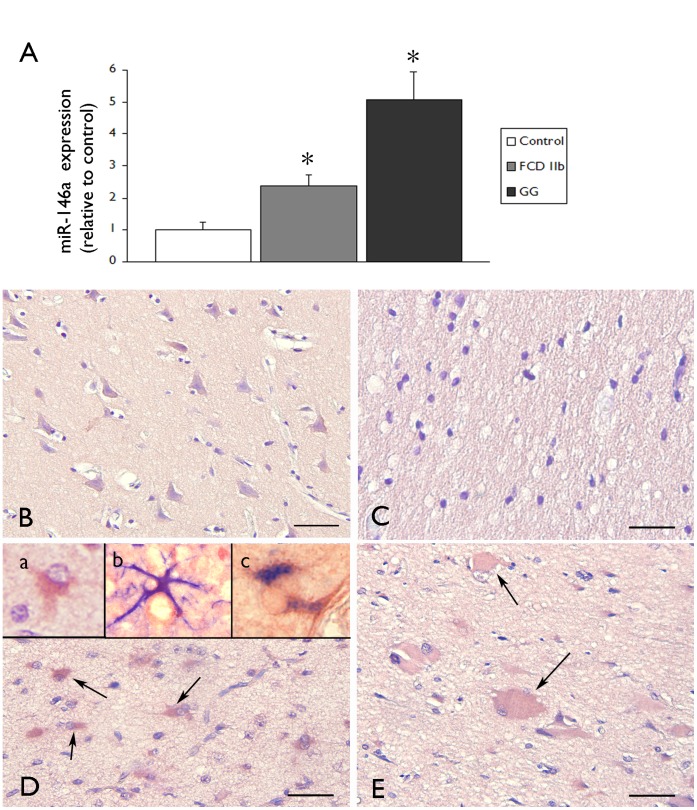
miR-146a expression in GG and FCD type IIb. (**A**) Quantitative real-time PCR of miR-146a in control cortex (n = 6; autopsy), FCD type IIb (n = 6) and GG (n = 5) specimens. miR-146a expression was normalized to that of the U6B small nuclear RNA gene (rnu6b). The error bars represent SEM; statistical significance: *P<0.05 compared to control. (**B–E**) In situ hybridization of miR-146a expression in control (B–C) and FCD type IIb (D–E) specimens. miR-146a was expressed at low levels in neurons and undetectable in glial cells in control grey (B) and white matter (C) specimens. Panels D: FCD type IIb tissue showing miR-146a expression in glial cells (arrows; insert a); inserts (b,c) in D show colocalization (purple) of miR-146a (red) in GFAP (blue) positive astrocytes. Panel E: FCD type IIb tissue showing miR-146a expression in balloon cells (arrows). Scale bar in B: B: 80 µm. C–E: 40 µm.

miR-146a expression was studied using qPCR in control human cerebral cortex samples and in GG and FCD type IIb samples. Expression of miR-146a was significantly increased in GG and FCD specimens compared to control cortex (Fig. 1A).

There were no significant differences in miR-146a expression between autopsy and surgical control samples (Figure S1). The cellular distribution of miR-146a in control cortex and FCD type IIb specimens was investigated using in situ hybridization (Fig. 1 B–E). miR-146a was undetectable in glial cells in control grey and white matter specimens (Fig. 1 B–C). In FCD specimens miR-146a expression was detected in glial cells with typical astroglia morphology, particularly in the areas of prominent gliosis (Fig. 1 D). Balloon cells displayed also miR-146a expression (Fig. 1 E). Double labelling confirmed miR-146a expression in GFAP-positive reactive astrocytes (Fig. 1 D, inserts), whereas no detectable expression was observed in HLA-DR positive cells of the microglial/macrophage lineage (not shown). miR-146a expression in reactive astrocytes was also observed in tissue specimens from patients with viral encephalitis and in tumor astrocytes in GG specimens (not shown).

### Regulation of miR-146a Expression by IL-1β in Human Glial Cells in Culture

**Figure 2:**
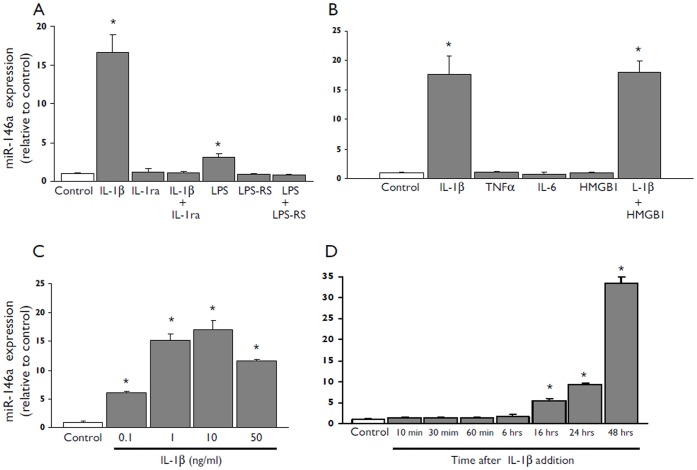
miR-146a expression levels in U373 glioblastoma cell line after exposure to IL-1β. Quantitative real-time PCR of miR-146a expression in U373 cells in culture. (**A**) Expression levels of miR-146a 24 hours after exposure to IL-1β (10 ng/ml) or LPS (100 ng/ml) in the presence or absence of the IL-1β receptor antagonist (IL-1ra; 1 µg/ml) or LPS-RS (10 µg/ml) respectively. (**B**) Expression levels of miR-146a 24 hours after exposure to IL-1β (10 ng/ml), TNFα (1 ng/ml), IL-6 (10 ng/ml), HMGB1 (40 nM alone or in the presence of IL-1β). (**C**) Expression levels of miR-146a 24 hours after exposure to 0.1, 1, 10 or 50 ng/ml of IL-1β. (**D**) Expression levels of miR-146a in U373 cells incubated for different durations (10, 30, 60 min and 6, 16, 24, 48) hours in the presence of IL-1β (10 ng/ml). Data are expressed relative to the levels observed in unstimulated cells and are mean ± SEM from two separate experiments performed in triplicate (*p<0.05 compared to control).

**Figure 3:**
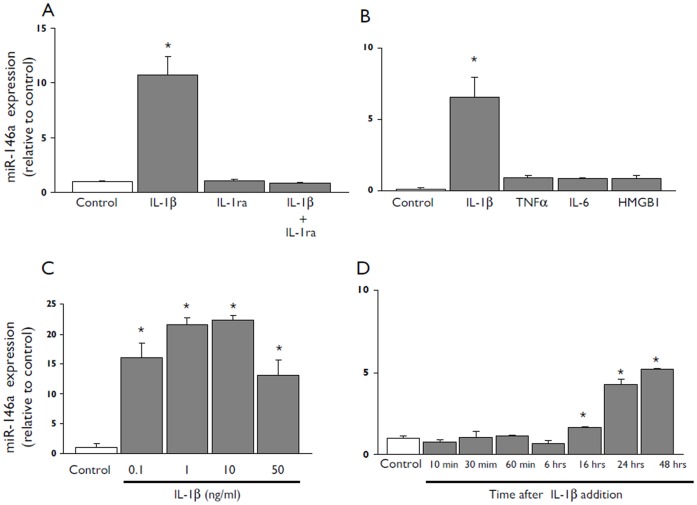
miR-146a expression levels in cultured human astrocytes after exposure to IL-1β. Quantitative real-time PCR of miR-146a expression in human fetal astrocytes in culture. (**A**) Expression levels of miR-146a 24 hours after exposure to IL-1β (10 ng/ml) or LPS (100 ng/ml) in the presence or absence of the IL-1β receptor antagonist (IL-1ra; 1 µg/ml) or LPS-RS (10 µg/m) respectively. (**B**) Expression levels of miR-146a 24 hours after exposure to IL-1β (10 ng/ml), TNFα (1 ng/ml), IL-6 (10 ng/ml), HMGB1 (40 nM). (**C**) Expression levels of miR-146a 24 hours after to 0.1, 1, 10 or 50 ng/ml of IL-1β. (**D**) Expression levels of miR-146a in cells incubated for different times (10, 30, 60 min and 6, 16, 24, 48) hours in the presence of IL-1β (10 ng/ml). Data are expressed relative to the levels observed in unstimulated cells and are mean ± SEM from two separate experiments performed in triplicate (*p<0.05 compared to control).

In the present study we used both U373 glioblastoma cell line and human fetal astrocytes in culture to examine the effect of IL-1ß and other inflammatory molecules on the expression levels of miR-146a. qPCR demonstrated that exposure to IL-1ß consistently increased the miR-146a expression in both cells types (Fig. 2 and 3). The effect of IL-1ß was blocked by the IL-1ß ra, a naturally occurring antagonist of the IL-1 receptor ([36]; Fig. 2 A and Fig. 3 A). Induction of miR-146a (although to a lower extent compared to IL-1ß) was also observed with LPS and blocked by LPS-RS (Fig. 2 A). In contrast, under our culture conditions we did not observe increased miR-146a levels after exposure to IL-6 (10 ng/ml), TNFα (1 ng/ml;) or HMGB1 (40 nM; Fig. 2 B and Fig. 3 B). IL-1ß induced upregulation of miR-146a at 0.1 ng/ml and its effect was maximal with doses ranging from 1 to 50 ng/ml (Fig. 2 C and Fig. 3 C); increased miR-146a levels were detected 16 hours after exposure to IL-1 ß (Fig. 2 D and Fig. 3 D).

### Regulation of miR-146a Expression by Transfection with Anti-miR-146a LNA or miR-146a Mimic

**Figure 4:**
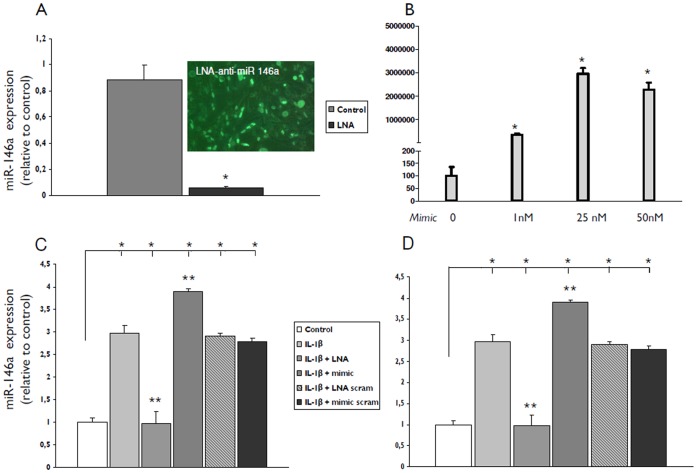
miR-146a expression levels after transfection with anti-miR-146a LNA or miR-146a mimic (pre-miR146a). Quantitative real-time PCR of miR-146a. (**A**) miR-146a expression after transfection with LNA-antimiR-146a (50 nM) in U373 cells; insert shows in green tranfected cells (**B**) miR-146a expression after transfection of miR-146a mimic (pre-mir-146a, 1, 25 and 50 nM). (**C**) miR-146a expression 24 hours after exposure to IL-1β in U373 cells transfected with LNA-antimiR-146a (50 nM) or miR-146a mimic (pre-mir-146a, 50 nM). (D) miR-146a expression 24 hours after exposure to IL-1β in cultured human astrocytes transfected with LNA-antimiR-146a (50 nM) or miR-146a mimic (pre-mir-146a, 50 nM). Data are expressed relative to the levels in unstimulated cells and are mean ± SEM from two separate experiments performed in triplicate (*p<0.05 compared to control; **p<0.05 LNA or mimic transfected cells stimulated with IL-1β, compared to IL-1β alone).

After transfection with 50 nM LNA-antimiR-146a into U373 glioblastoma cell line (Fig. 4 A) and human fetal astrocytes in culture (not shown) for 24 hours, qPCR revealed significant reduction of miR-146a. On the other hand significant overexpression of miR-146a was seen in U373 glioblastoma cell line (Fig. 4 B) and human fetal astrocytes in culture (not shown) after transfection with miR-146a mimic (pre-miR-146a) for 24 hours at concentrations ranging from 1 to 50 nM. Upon stimulation with 10 ng/ml IL-1ß for 24 hours lower miR-146a levels were observed in cells transfected with LNA-anti miR-146a (but not with the LNA-control) and higher levels in cells transfected with miR-146a mimic (but not by the mimic-control) compared to non-transfected cells (Fig. 4 C and D).

### Effects of miR-146a Inhibition or Overexpression on its Downstream Targets IRAK-1, IRAK-2 and TRAF-6

**Figure 5:**
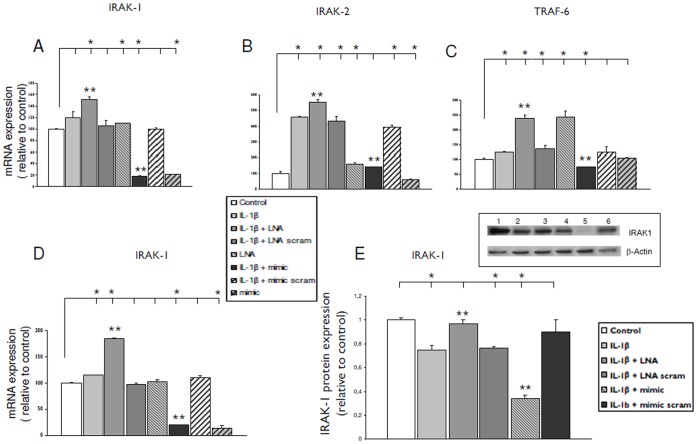
Expression levels of the miR-146a targets (IRAK-1, IRAK-2 and TRAF-6) after transfection with anti-miR-146a LNA or miR-146a mimic. (**A–C**) Quantitative real-time PCR of IRAK-1 (A), IRAK-2 (B) and TRAF-6 (C) expression 24 hours after exposure to IL-1β in U373 glioblastoma cell line transfected with LNA-antimiR-146a (25 nM) or miR-146a mimic (pre-mir-146a, 50 nM). (**D**) Quantitative real-time PCR of IRAK-1, 24 hours after exposure to IL-1β in cultured human astrocytes transfected with LNA anti-miR-146a (50 nM) or miR-146a mimic (pre-mir-146a, 50 nM). Data are expressed relative to the levels in unstimulated cells and are mean ± SEM from two separate experiments performed in triplicate (**E**) IRAK-1 protein expression 24 hours after exposure to IL-1β in glial cells transfected with LNA anti-miR-146a (50 nM) or miR-146a mimic (pre-mir-146a, 50 nM); Representative immunoblot (1 control; 2, IL-1β; 3, IL-1β + LNA-antimiR-146a; 4, IL-1β + LNA-antimiR-146a scramble; 5, IL-1β + mimic; 6, IL-1β + mimic scramble) and optical density measurements. Data are expressed relative to the levels in unstimulated cells and are mean ± SEM from two separate experiments (*p<0.05, compared to control; **p<0.05, LNA or mimic transfected cells stimulated with IL-1β compared to IL-1β alone).

Transfection with LNA-anti miR-146a or miR-146a mimic modulated the mRNA expression levels of downstream targets of mir-146a, such as IRAK-1, IRAK-2 and TRAF-6 after exposure to IL-1ß in both U373 (Fig. 5 A–C) and fetal astrocytes (Fig. 5 D, IRAK-1; IRAK2 and TRAF-6: data not shown). In particular, a consistent downregulation of all 3 targets was observed under conditions in which miR-146a was overexpressed. Increased mRNA levels of IRAK-1, IRAK-2 and TRAF-6 were observed after transfection with LNA-anti miR-146a (Fig. 5 B–C, U373; Fig. 5 D, IRAK-1, fetal astrocytes). Western blot analysis showed the downregulation of IRAK-1 protein induced by IL-1ß in both U373 (Fig. 5 E) and fetal astrocytes (not shown). This effect was enhanced by transfection with miR-146a mimic. In contrast LNA-anti miR-146a transfection resulted in the restoration of IRAK1 protein to control level. No significant differences in IRAK-2 and TRAF-6 proteins were observed in either cell culture.

### Effects of miR-146a Inhibition or Overexpression on IL-1β Mediated Induction of IL-6 and COX-2

**Figure 6:**
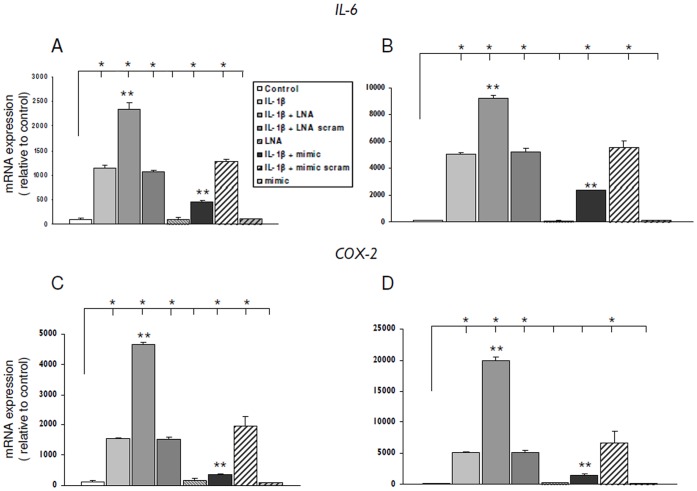
Effect of anti-miR-146a LNA or miR-146a mimic upon IL-1β-induced IL-6 and COX-2 mRNA. Quantitative real-time PCR of IL-6 (A–B) and Cox-2 (C–D). (**A–B**) IL-6 mRNA levels, 24 hours after exposure to IL-1β in U373 cells (A) and cultured human astrocytes (B) transfected with LNA-antimiR-146a (50 nM) or miR-146a mimic (pre-mir-146a, 50 nM). (**C–D**) COX-2 mRNA levels, 24 hours after exposure to IL-1β in U373 cells (C) and cultured human astrocytes (D) transfected with LNA-antimiR-146a (50 nM) or miR-146a mimic (pre-mir-146a, 50 nM). Data are expressed relative to the levels in unstimulated cells and are mean ± SEM from two separate experiments performed in triplicate (*p<0.05 compared to control; **p<0.05, LNA or mimic transfected cells stimulated with IL-1β compared to IL-1β alone).

IL-1ß induced expression of two major inflammatory mediators, IL-6 and Cox-2 was modulated by transfection of glial cells with LNA-anti miR-146a or miR-146a mimic. Transfection with miR-146a mimic downregulated IL-1ß induced IL-6 and Cox-2 mRNA levels in both U373 glioblastoma cells and human fetal astrocytes in culture (Fig. 6 A–D). In contrast, LNA anti-miR-146a transfection significantly increased the levels of IL-6 and Cox-2 induced by IL-1ß (Fig. 6 A–D).

**Figure 7:**
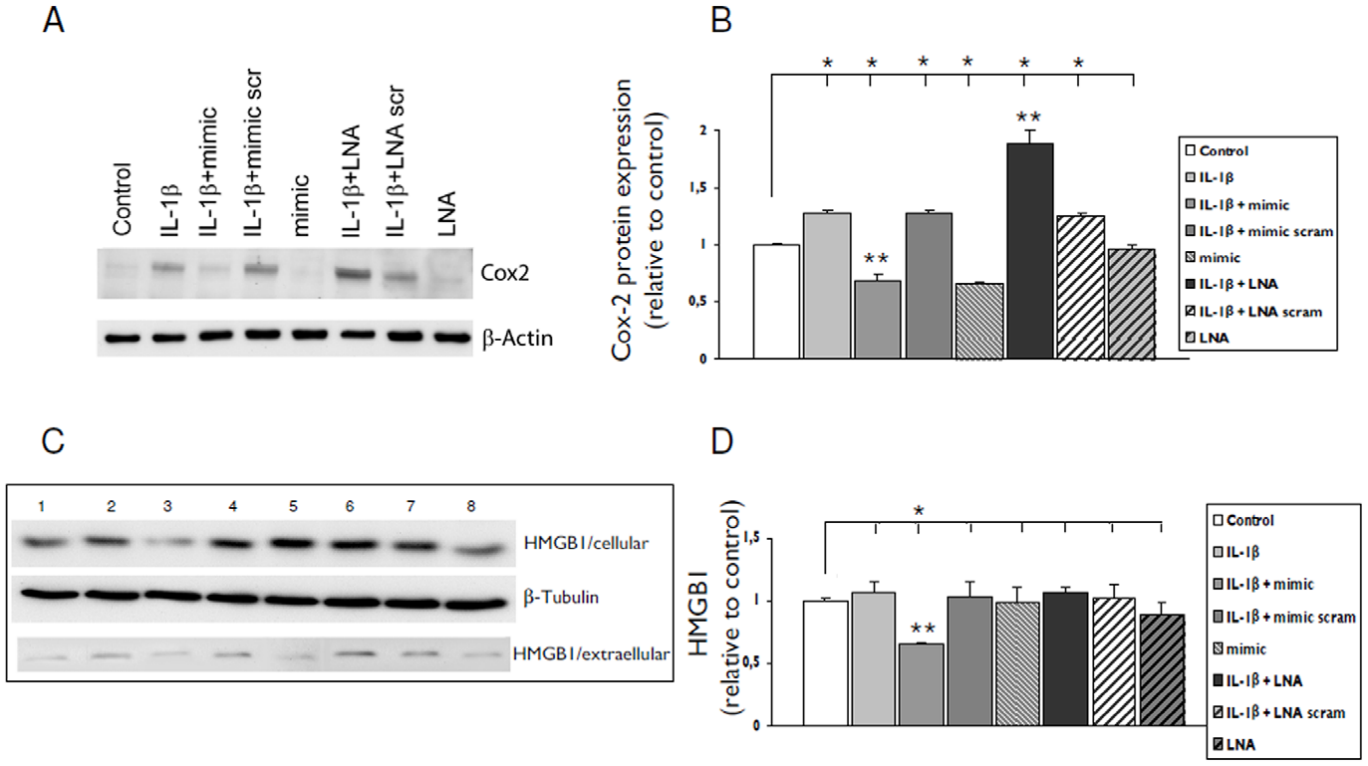
Effect of anti-miR-146a LNA or miR-146a mimic upon IL-1β-induced COX-2 protein and release of HMGB1. COX-2 protein expression 24 hours after exposure to IL-1β in U373 cells transfected with LNA-antimiR-146a (50 nM) or miR-146a mimic (pre-mir-146a, 50 nM). (**A**) Representative immunoblot and (**B**) densitometric analysis: values (optical density units relative to the optical density of β-actin) are mean ± SEM of two separate experiments performed and are expressed relative to the levels in unstimulated cells. (**C**) HMGB1 immunoblot (1 control; 2, IL-1β; 3, IL-1β + mimic; 4, IL-1β + mimic scramble; 5 mimic; 6, IL-1β + LNA-antimiR-146a; 7, IL-1β + LNA-antimiR-146a scramble 8, LNA) and densitometric analysis (**D**, optical density units of cellular HMGB1 relative to the optical density of β-actin). *p<0.05, compared to control; **p<0.05, LNA or mimic transfected cells stimulated with IL-1β compared to IL-1β alone.

Western blot analysis confirmed the different effects of LNA-anti-miR-146a and miR-146a mimic on COX-2 protein expression in IL-1ß stimulated cells; transfection with miR-146a mimic reduced whereas LNA-anti-miR-146a increased the COX-2 expression (Fig. 7 A–B).

### Effects of miR-146a Inhibition or Overexpression on IL-1β Mediated Release of Inflammatory Mediators

We evaluated the release of high mobility group box (HMGB1; by western blot analysis) and the release of MCP1 (by ELISA) in response to IL-1ß stimulation in both U373 and fetal astrocytes. Transfection of U373 with miR-146a mimic decreased both the cellular and extracellular levels of HMGB1 (Fig. 7 C–D) compared to IL-1ß stimulated cells. The release of MCP-1 induced by IL-1ß was negatively regulated by transfection with miR-146a mimic (MCP-1 pg/ml: control: 1582±193; IL-1ß: 27151±1368; IL-1ß + miR-146a mimic: 11054±2027; p<0.05 compared to IL-1ß stimulated cells), whereas transfection with LNA-anti miR-146a did not significantly affect the release induced by IL-1ß.

**Figure 8:**
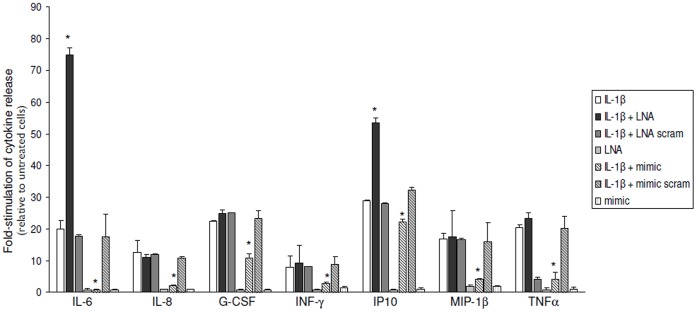
Effect of anti-miR-146a LNA or miR-146a mimic upon IL-1β-induced release of inflammatory molecules. (**A**) Cytokine release 24 hours after exposure to IL-1β in U373 cells transfected with LNA-antimiR-146a (50 nM) or miR-146a mimic (miR-146a mimic, 50 nM). Data are expressed relative to unstimulated control cells (mean ± SEM from three separate experiments). In comparison with IL-1ß alone, cultures stimulated with IL-1ß and transfected with LNA-anti miR-146a exhibited significant increase of IL-6 and IP-10 release, whereas transfection of glial cells with miR-146a mimic significantly decreased the levels of IL-6, IL-8, G-CSF, IFN-γ, IP-10, MIP-1β, and TNF-α (* p<0.05). LNA-anti miR-146a and miR-146a mimic alone did not significantly affect the levels of cytokines in the culture medium, compared to non treated cells.

The release of 16 cytokines/chemokines was evaluated using the human magnetic 10-plex panel in U373 glioblastoma cell line. IL-5, IL-17 and IFN-α2 were under detection limits in our culture conditions. The amount of IL-10, IL-13, IL-18 and VEGF did not significantly change after the different treatments. IL-1β significantly increased the release IL-6, IL-8, IFN-γ, IP-10, MIP-1β, G-CSF, Rantes and TNF-α (Fig. 8). Transfection with LNA-anti miR-146a before treatment with IL-1β, significantly increased the levels of IL-6 and IP-10; whereas transfection with miR-146a mimic before treatment with IL-1β significantly decreased the levels of IL-6, IL-8, G-CSF, IFN-γ, IP-10, MIP-1β, and TNF-α (Fig. 8).

## Discussion

The miR-146a has recently been shown to be upregulated in experimental models of epilepsy, as well as in human TLE. [20], [21]. In a rat model of TLE strong upregulation was detected in astrocytes 1 week after status epilepticus (during epileptogenesis) which corresponds to the latent period and the time of maximal astroglial activation and upregulation of various genes involved in the immune response [37], [38]. These observations are in line with other studies supporting the association between this specific miRNA and human inflammatory diseases [15], [16]. In particular, upregulation of miR146a has been detected in active multiple sclerosis lesions [39] and in human Alzheimer disease (AD) brain [19], suggesting a key role of this miRNA in governing astrocyte activation and function in both pathologies. However, there is still little information regarding miR-146a function in astrocytes, in particular regarding the effect on astroglia mediated inflammatory pathways in relation to epilepsy.

In the present study, we demonstrate the overexpression of miR-146a in glioneuronal lesions from patients with medically intractable epilepsy and provide evidence for a role of miR-146a in the regulation of the astroglia mediated inflammatory response.

### MiR-146a Expression in GG and FCD IIB

GG and FCD type IIb represent a major cause of drug-resistant epilepsy [23], [25] and are characterized by a prominent activation of various inflammatory pathways, including the TIR signaling pathway [9], [40]–[43]. In the present study, we provide evidence for upregulation of miR-146a in these epilepsy-associated glioneuronal lesions. Increased levels of miR-146a were detected GG and FCD type IIb compared to both control autopsy material and histologically normal surgical cortex (without alteration in cortical lamination or astrogliosis) from patients with epilepsy. The latter excludes the direct effect of recurrent seizures and antiepileptic drugs on the detected expression level of miR-146a in FCD specimens. Thus seizures alone may not account for changes in miR-146a expression. The absence of differences in miR-146a expression between autopsy and surgical specimens excludes the possibility that this is an artefact of autopsy delay. Moreover, recent studies indicate a robust stability of miRNAs, supporting the accuracy of miRNA measurements with RT-qPCR [44]. *In situ* hybridization confirmed the increased expression of miR-146a in reactive astrocytes which are abundantly present within the dysplastic cortex in FCD IIb. This observation suggests an important role of miR-146a in astrocytes in epilepsy associated lesions. Expression of miR-146a was also detected in balloon cells. Since balloon cells have been shown to represent a major source of proinflammatory molecules and possibly contribute to the TIR signaling [9], [41], [45], further evaluation of the miR-146a function in these cell types could be performed in recently described culture system [46]. Evaluation of the function of this miRNA in long-term epilepsy associated glioneuronal tumors and in malformations of cortical development (containing balloon cells with a stem cell phenotype), is particularly interesting in view of the recently described function of miR-146a in modulating neural stem cell proliferation and differentiation through regulation of the key neural stem cell factor Notch1 [47]. Further investigation is also needed to evaluate the levels of different miR-146a targets in GG and FCD.

### miR-146a is Regulated in Human Glial Cells in Culture by IL-1β

Since miR-146a was up-regulated in astrocytes in the epileptogenic lesions (FCD, present results; TLE [20], [21]) the expression of miR-146a was further analyzed in culture, using U373 glioblastoma cell line and human fetal astrocytes. We evaluated the expression levels of miR-146a upon to exposure to inflammatory molecules (IL-1β, IL-6, TNFα or HMGB1) that are known to be up-regulated in epileptogenic tissue from FCD and TLE patients; (for review see [3]) and to the TLR4 ligand LPS. Both cell line and primary astrocytes were found to exhibit a prominent upregulation of miR-146a expression in response to IL-1β stimulation. In contrast, LPS induced modest up-regulation whereas IL-6, TNFα and HMGB1 were not effective in inducing significant changes in miR-146a expression. The effect of IL-1β in human astrocytes is in line with recent studies in culture [39], [48], supporting an important role of this cytokine in the regulation of astroglial miR-146a expression. The effect of IL-1β was concentration and time dependent and was observed from 16 hours after exposure to the cytokine. The observation that IL-1Ra inhibited the effect of IL-1β on miR-146a is consistent with the fact that IL-1β signals through the type I IL-1β receptor which is upregulated in epileptogenic lesions [[49]; for review see (Aronica, *et al.*, 2011; Aronica, *et al.* in press)].

### Regulation of IL-1β Pathway by miR-146a

To further investigate the function of miR-146a in human glial cells, we studied the effects of overexpression (with miR-146a mimic) or blockade of miR-146a (with antisense miR-146a; LNA- anti-miR-146a) on the expression levels of downstream signaling molecules (IRAK-1, IRAK-2 and TRAF-6) of the TIR signaling pathway that have previously been shown to be miR-146 targets [15], [17]–[19]. In both the glioblastoma cell line and human astrocytes transfection with miR-146a mimic, significantly reduced IRAK-1, IRAK-2 and TRAF-6 mRNA and IRAK-1 protein after stimulation with IL-1β. In a previous study in primary cultures of human astroglial cells exposed to IL-1β and Aβ42 peptide, it has been shown that an antisense miRNA-146a inhibits miRNA-146a and controls IRAK-1, but not IRAK-2 expression, suggesting an independent regulation of the two targets under these experimental conditions [19]. However, in our study, both IRAK-1 and IRAK-2 mRNA were positively regulated by LNA-anti-miR-146a (and negatively by miR-146a mimic) in IL-1β stimulated human astrocytes. This observation argues against a proinflammatory role of this miRNA-146a, as result of a compensatory upregulation of IRAK2 (at least in the absence of Aβ42 peptide). However, the results at the protein level may suggest a preferential regulation of IRAK-1, which is prominently downregulated by miR-146a mimic. The effect of miR-146a on multiple targets may also vary in different cell types and upon different stimuli. For example, it has been shown that miR-146a is upregulated during viral infection in macrophages and acts as a negative regulator of the retinoic acid-inducible gene I (RIG-I)-like helicases by targeting not only IRAK1 but also TRAF6 and IRAK2 [50].

### miR-146a Regulates the Expression IL-6 and COX-2

To further evaluate the function of miR-146a in response to inflammatory stimuli, we investigated the effect of miR-146a overexpression or reduction on IL-6 and COX-2, two major inflammatory molecules induced by IL-1β [for reviews see [51], [52]]. Astrocytes are a major source of IL-6 and prominent induction of this cytokine on IL-1β stimulation has been previously reported in human fetal astrocytes in culture [53], [54]. Both IL-6 and COX-2 have been reported to be associated with astrogliosis and activation of the innate immune response in different pathological conditions (for reviews see [3], [51], [52]). Furthermore, miR-146a has a complementarity with the COX-2 3′ UTR and miR-146a mimic has been recently shown to reduce COX-2 mRNA levels in fibroblasts [55] and glial cells [56].

The *in vitro* overexpression and knock-down studies showed that transfection with miR-146a mimic reduces the IL-6 and COX-2 mRNA levels in cultures stimulated with IL-1β, whereas LNA-anti-miR-146a had the opposite effect. Western blot analysis further showed that ectopic miR-146a caused a reduction of both basal and IL-1β stimulated expression of COX-2 protein. Conversely, an upregulation of the COX-2 protein was observed after knockdown of miR-146a with LNA-anti-miR-146a in IL-1β -treated cells. The anti-inflammatory role of miR-146a is also supported by its ability to regulate the IL-1β induced release of several other proinflammatory factors, such as IL-6, IL-8, G-CSF, IFN-γ, IP-10, MIP-1β, and TNF-α, HMGB1 and MCP1.

Although IL-1ß increased miR-146a levels, these levels were not sufficient to significantly reduce the expression of the miR-146a targets studied. However, we cannot exclude a physiological control on the levels that could be reached in the absence of miR-146a production. Accordingly, transfection with LNA in the presence of IL-1ß which blocked the effect of miR-146a, further increased IRAK-1 and IRAK-2 mRNA, as well as IL-6 and COX-2 mRNA and protein levels *in vitro*. The effect of mimic in reducing the target expression indicates that overexpression is required to exert an anti-inflammatory effect. A recent study indicates that transcript sequestration and degradation via miRNA-mRNA complex formation is a complex and dynamic process and that miRNA synthesis rate is the dominant control element that has to be taken into consideration in the development of miRNA based therapies [57].

Our observations, together with other emerging data, suggest a role for a miR-146a-mediated regulation of inflammation in glial cells and provide an opportunity to develop novel therapeutic strategies in neurological disorders associated with chronic inflammation and immune deregulation.

## Supporting Information

Figure S1 **miR-146a expression in control cortex.** Quantitative real-time PCR of miR-146a in control cortex (autopsy: control 1 and surgical tissue: control 2). miR-146a expression was normalized to that of the U6B small nuclear RNA gene (rnu6b).
(TIF)

Table S1 **Summary of clinical findings of epilepsy patients and controls.** FCD = Focal Cortical Dysplasia. GG: ganglioglioma; M = male; F = female.
(DOC)
