# Supplementary figures and images for: MicroRNA-146a: A Key Regulator of Astrocyte-Mediated Inflammatory Response
Source: PLoS One. 2012 Sep 13;7(9):e44789. doi: 10.1371/journal.pone.0044789 (PMC3441440; doi:10.1371/journal.pone.0044789)

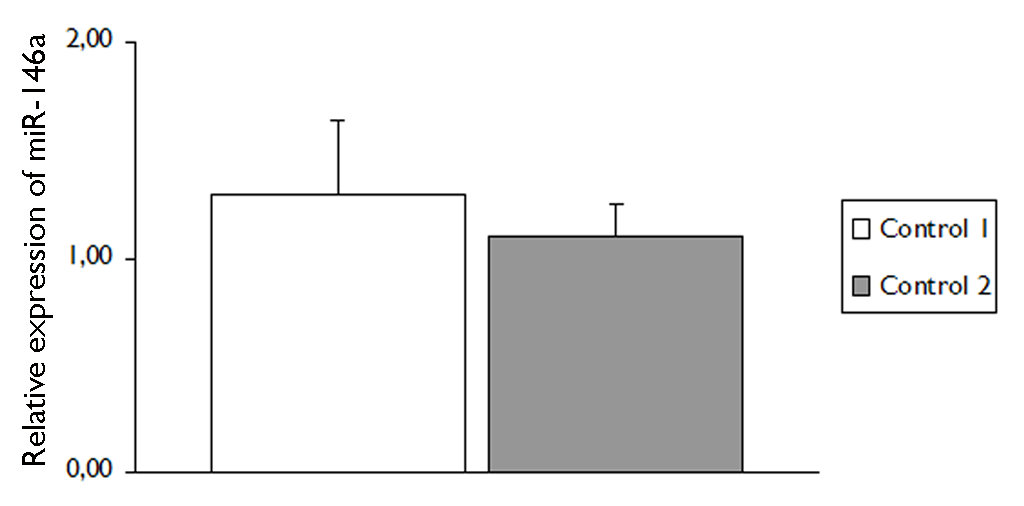

Supplement: Figure S1 — miR-146a expression in control cortex. Quantitative real-time PCR of miR-146a in control cortex (autopsy: control 1 and surgical tissue: control 2). miR-146a expression was normalized to that of the U6B small nuclear RNA gene (rnu6b). (TIF) [file pone.0044789.s001.tif]
