# Supplementary material for: MicroRNA-146a: A Key Regulator of Astrocyte-Mediated Inflammatory Response
Source: PLoS One. 2012 Sep 13;7(9):e44789. doi: 10.1371/journal.pone.0044789 (PMC3441440; doi:10.1371/journal.pone.0044789)
Supplement: Table S1 — Summary of clinical findings of epilepsy patients and controls. FCD = Focal Cortical Dysplasia. GG: ganglioglioma; M = male; F = female. (DOC) [file pone.0044789.s002.doc]

**Table S1.** Summary of clinical findings of epilepsy patients and controls.

| **Pathology type** | **Number of cases** | **Gender**  **(M/F)** | **Mean age at surgery (years/range)** | **Localization** | **Mean duration of epilepsy (years/range)** |
| --- | --- | --- | --- | --- | --- |
| FCD IIb | 6 | 3/3 | 25.5 (21-33) | 3 Temporal  3 Frontal | 11.1 (6-17) |
| GG | 5 | 3/2 | 29. 5 (16-34) | Temporal | 10.1 (3-16) |
| Controls  (no epilepsy/autopsy ) | 6 | 3/3 | 40.3 (25-52) | Temporal | - |
| Controls  (no FCD/surgical) | 3 | 3/0 | 33.0 (22-36) | Temporal | 13.0 (10-18) |

FCD = Focal Cortical Dysplasia. GG: ganglioglioma; M = male; F = female.

**Figure S1****. miR-146a expression in control cortex**

Quantitative real-time PCR of miR-146a in control cortex (autopsy: control 1 and surgical tissue: control 2). miR-146a expression was normalized to that of the U6B small nuclear RNA gene (rnu6b).
